# The impact of predators and vegetation on shoaling in wild zebrafish

**DOI:** 10.1098/rsos.240760

**Published:** 2024-09-25

**Authors:** Ishani Mukherjee, Anuradha Bhat

**Affiliations:** ^1^ Department of Biological Sciences, Indian Institute of Science Education and Research Kolkata, Mohanpur, West Bengal 741246, India

**Keywords:** behavioural plasticity, group behaviour, wild zebrafish, predation, vegetation

## Abstract

In their natural habitats, animals experience multiple ecological factors and regulate their social responses accordingly. To unravel the impact of two ecological factors on the immediate behavioural response of groups, we conducted experiments on wild zebrafish shoals in arenas with vegetation, predator cues, and both factors simultaneously or neither (control treatments). Analysis of 297 trials revealed that while shoals formed significantly larger subgroups in the presence of predator cues, their subgroup size was comparable to control treatments when they faced predator cues and vegetation. Shoals were highly polarized in open arenas, in the absence of either ecological factors and in the presence of predator cues (with/without vegetation). The presence of vegetation alone, however, significantly reduced shoal polarization. Furthermore, food intake was significantly reduced when predator cues and/or vegetation were present. Tracking individuals revealed that (i) individuals within shoals receiving predator cues had a significantly higher probability to continue being in a group compared with control treatments and (ii) individuals occupying the front positions deviated less from their median position within a shoal as compared with other individuals regardless of predator cues. The adaptability of animals depends on behavioural responses to changing environments, making this study significant in the context of environmental changes.

## 1. Introduction

Across taxa, ecological factors such as predation, vegetation, resource availability and habitat complexity shape behaviour [1–5]. Behavioural changes indirectly control a variety of large-scale ecological functions such as nutrient cycling, primary productivity, pathogen transfer, inter-species interactions and habitat provision [6]. Although variations in ecological factors over evolutionary timescales elicit long-term behavioural changes [7,8], sudden changes in these factors can trigger immediate behavioural responses. Immediate behavioural changes are reversible, can determine an individual’s success or adaptability in a new environment and, consequently, often guide evolutionary change [9]. As one of the quickest responses to environmental shifts, behavioural changes are crucial in determining an animal’s success in a changing environment, making their study essential.

In the wild, behavioural responses elicited by animals to environmental changes are dependent on a multitude of ecological variables. To comprehend an animal’s immediate responses to multiple factors, it is essential to grasp how ecological factors, either individually or in combination, exert distinct influences on behaviour. In the present study, using a shoaling cyprinid, the zebrafish (*Danio rerio*), we gain insight into immediate group-level behavioural response to changes in their habitat. We examine the immediate shoal responses of wild-caught zebrafish towards two key ecological factors: predation and vegetation cover.

The influence of predation and vegetation cover has been studied on several terrestrial taxa. For instance, in lions (*Panthera leo*), males rely on the ambush hunting strategy, and therefore to successfully hunt heavily rely on dense vegetation [10]. In the case of fox squirrels (*Sciurus niger*), the role of vegetation structure on anti-predator behaviour is dependent on the type of predators [11]. The water-drinking behaviour and frequency in red colobus monkeys (*Procolobus kirkii*) are significantly influenced by the time spent by them in mangroves [12]. Studies have long examined anti-predator responses, such as heightened group cohesion and increased group size (safety in numbers), heightened vigilance and improved predator confusion, as strategies to reduce individual vulnerability to attacks [13,14]. For instance, elks (*Cervus elaphus*) move into protective cover (timber) in response to wolf presence [15]. Meerkats (*Suricata suricatta*) produce several discrete call types to convey changes in predation risk [16].

In aquatic habitats too, behaviour is greatly influenced by both predation and vegetation cover. For instance, in fishes, studies have shown that predation strongly shapes social interactions and shoal properties within shoals [17–19]. The predator avoidance strategy in golden shiners (*Notemigonus crysoleucas*) is dependent on the attack strategy of their predator [20]. In other fish species, the presence of vegetation, on the other hand, decreases prey capture, swimming speed and shoaling tendencies [21,22].

Here, we aim to gain insight into the behavioural plasticity exhibited by shoals in response to ecological variables. We recorded the immediate group-level changes in the presence of vegetation and/or predation, we recorded the responses of wild zebrafish shoals in the presence of these ecological variables. In shoaling fishes, predator avoidance responses and their ability to forage are directly linked to their survival [23–26], and hence we examine these behaviours in response to vegetation/predation pressure. Previous literature suggests that fish shoals adhere to safety in numbers in the presence of a predator [14,27,28]. Shoal polarization or the alignment of shoal members in a common direction (exhibited for coordinated motion) is known to be disrupted in the presence of a predator. When shoals encounter a predator, shoal polarization either increases or decreases depending on the species [19,20,29–36].

Wild zebrafish occurring in freshwater streams along the Gangetic drainage in India experience variable predation risk, ranging from moderate to high dependent on habitat and vegetation characteristics (personal observation). These habitats frequently undergo dynamic changes, with temporal and/or spatial fluctuations in vegetation and predation pressure, influenced by factors such as seasonality or anthropogenic alterations. Hence, zebrafish shoals would be likely to exhibit notable plasticity in their shoaling properties as responses to variations in these ecological factors. We hypothesized that when individuals are exposed to sudden environmental changes in the form of predator cues they would (i) form large, polarized or polarized groups and (ii) move away less from the group. In the wild, zebrafish are known to shoal among vegetation (personal observation, [37–41]), and therefore it is likely that vegetation plays an important role in shaping anti-predator responses in zebrafish. We speculated that in the presence of vegetation, individuals would take refuge underneath vegetation—a common anti-predator response in fishes [39–42]. Furthermore, we also hypothesized that foraging (food intake) would reduce (i) in the presence of predator cues as individuals would engage in anti-predator behaviour (over foraging) and (ii) in the presence of vegetation as there would be a reduction in visual information on the presence of food.

## 2. Methods

Wild zebrafish shoals were collected from shallow water bodies on the Ganges drainage basin in West Bengal in December 2019 and January 2020 (habitat specifics detailed in electronic supplementary material, §S1). Shoals were brought to the laboratory and were maintained in bare, aerated 60 l tanks filled with aged filtered tap water. Shoals were maintained at a density of 100–120 individuals per tank. Four *Channa* spp. (snakeheads) individuals were also collected from the same habitat (mean length: 12 cm), brought to the laboratory and were kept in 18 l tanks (one individual per tank). A temperature range of 23–25°C and a constant lighting condition of 12 h dark : 12 h light in the laboratory were maintained. While the zebrafish were fed daily ad libitum with freeze-dried bloodworms or brine shrimp (*Artemia* spp.), the snakeheads were fed daily with pellet food or zebrafish that died of natural causes.

### 2.1. Experiments

Shoals were gently introduced into a 75 cm × 75 cm ×12 cm tank and were recorded for 20 min under the following four treatments. (i) Control treatments (CT) in which shoals were placed in an arena without predator cues or vegetation. (ii) Predator treatments (PT) in which, following previous studies, olfactory cues from their natural predator (6.5 l of tank water collected from a *Channa* tank) were gradually added to the arena centre [43]. Previous studies show that water from a tank housing a predator contains olfactory cues from the predator that evoke anti-predator responses in prey [44–47]. Thus, water from a *Channa* tank was added in treatments simulating the presence of a predator. Prior control experiments conducted in the laboratory have established that the gentle addition of water into the arena centre had no impact on shoaling properties (electronic supplementary material, figure S1). Prey species elicit anti-predator responses based on previous exposure to predators [48]. The test shoals were wild-caught and thus would recognize the danger of predator cues and elicit anti-predator responses. (iii) Vegetation treatments (VT) in which shoals were recorded in an arena with six identical aquarium plants, three on each diagonally opposed corner (two corners were devoid of vegetation). (iv) Predator and vegetation treatments (PVT) in which shoals were placed in an arena with vegetation, after which predator cues were gradually added.

Experiments were performed between 11.00 and 15.00. In their natural habitats, wild zebrafish typically form shoals with 10–20 individuals [49], and thus a shoal size of 10 individuals was maintained across all trials. Throughout the trials, the test arena maintained a consistent water depth of 5 cm. Shoals encountering predator cues were initially introduced into the arena with a water depth of approximately 4 cm. This initial depth was adjusted to reach 5 cm upon the addition of olfactory cues. To avoid the impact of sex of individuals on shoaling behaviour [50,51], we randomly chose individuals who constituted a shoal and thereby maintained the population sex ratio of a roughly equal number of males and females (as seen in natural populations). A 3.5 cm thermocol (polystyrene) sheet was placed under the arena to minimize ground vibrations. Two 20 W LED light bulbs on either side of the arena maintained a constant light source. Two minutes after the gradual addition of predator cues (to allow shoals to recover from disturbances—if any), or immediately after acclimatization in VT or CT, the shoal was video recorded for 20 min at 25 frames per second using an overhead camera (Canon Legria HF R306). Following this, 0.25 g of freeze-dried blood worms were introduced into the arena centre and the shoal was video recorded for another 5 min. The arena was emptied and rinsed with aged water between consecutive trials to remove cues from blood worms, from conspecifics or from a predator. Sixty unique shoals (30 across all treatments, 15 additional in PT and 15 additional in CT) were tested by performing 210 randomized trials (150 shoaling trials and 60 foraging trials). Each shoal was tested once per day and 6–8 trials were conducted daily. A shoal was tested across all four treatments in a randomized order over the course of four consecutive days. Between trials, each shoal was kept in a separate 25 l tank to maintain identity. A single observer (I.M.) blind to the treatment analysed the video recordings.

To extend the findings of the above experiment to field conditions, a follow-up experiment was performed in their natural habitat. In their natural habitats, predator cues may be less potent and occur with a variety of other cues. This study was conducted to compare the time taken for a shoal to emerge from underneath vegetation between treatments conducted in a controlled laboratory setup (in the absence of cues) and those carried out in the field, with the presence of various cues, including predator cues. The detailed experimental protocol for the follow-up study has been provided in electronic supplementary material, §S2.

### 2.2. Data preparation

From the video recording, shoaling behaviour was quantified by manually noting the size (number of individuals) of the largest subgroup and the polarization state of shoals every 30 s (750 frames) for 20 min. Individuals were regarded to be within a subgroup if any portion of their bodies was within two body lengths of another. The polarization score was calculated using the following as the proportion of the largest subgroup’s members aligned in a common direction [52,53], with an angle between −30° and +30° (manually calculated by inspecting each frame every 30 s on ImageJ [54]) and also as the mean heading vector of the whole group. We found the methods fetching comparable results (as detailed in electronic supplementary material, §S3). Owing to the fact that individuals in treatments with vegetation could not be tracked, we relied on the manual calculation to compare all four treatments. To check whether largest subgroup size and shoal polarization were consistent over time, the 20 min videos were divided into four 5 min sessions (sessions 1, 2, 3 and 4, in sequence of recording). Thereafter, the mean largest subgroup size and the mean polarization score for each session were calculated. The proportion of shoal underneath vegetation in VT and PVT treatments was determined every 30 s. To estimate foraging across the four treatments, the number of bites at bloodworms by each shoal in the first 2 min of the recording was counted.

To analyse temporal dynamics in shoals, individuals in CT and PT treatments were first tracked for the first 5 min (or 7500 frames) using idTracker. Thereafter, errors in their trajectories (if any) were manually corrected using an assisting software (idPlayer) to reach a tracking accuracy of almost 100% [55]. All individuals across 30 shoals (15 per treatment) were tracked to obtain 300 trajectories. Tracking in treatments with vegetation (VT and PVT) was not feasible as it was not possible to determine the precise position of fish underneath vegetation. Following Borner *et al*. [56] and Krause & Ruxton [57], we assigned solitary or group states to individuals every 10 s (250 frames). While individuals within four-body-length distance from other individuals were considered a group, individuals outside this zone were solitary. The rationale for setting the criteria of being within four body lengths to be in group state and setting the criteria of being within two body lengths to be a part of a subgroup is as follows: four body lengths is a looser cut-off and is optimum (as supported by other studies on schooling fish) to categorize whether an individual is within a group or is alone. On the other hand, two body lengths is a more stringent cut-off, and therefore was used to assign individuals into subgroups. The probability of not switching states, i.e. remaining solitary and remaining in group state, was calculated for each individual.

Next, following Doughty *et al*. [58], we manually noted down the movement order of individuals within the largest subgroup every 10 s (every 250 frames). The leader of the largest subgroup (of size *n*) received place 1, the individual spatially closest to the leader received position 2 and so forth. The last position was *n*. Following Fischhoff *et al.* [59], we normalized for variations in the largest subgroup size by calculating their order index using the following formula:


$$\text{Order index}=\frac{\left(\text{Position}\times 2\right)-1}{\text{Subgroup size}\times 2}.$$

The order index was calculated every 10 s across 5 min i.e. a total of 30 times. Thereafter, from multiple order indices, the median order index of individuals or the mid-value of the all order indices was calculated. The standard deviation from their median order indexes was calculated using the following formula:


$$\text{Standard deviation from median order index}=\sqrt{\frac{1}{30}\sum {\left|order\;index-median\;order\;index\right|}^{2}}.$$

### 2.3. Statistical analysis

All analyses were performed using R Studio [60]. Generalized linear mixed models (GLMMs) were built (using ‘lme4’ [61] and ‘lmerTest’ [62]) to understand the effect of (i) treatment (CT, PT, VT or PVT) and session (sessions 1–4) on largest subgroup size, (ii) treatment, session and size of subgroup (subgroups comprising more than five individuals were considered big and fewer than five individuals were considered small) on shoal polarization, and (iii) treatment on percentage shoal under vegetation. In these GLMMs, shoal identity was incorporated as the random factor. Similarly, separate generalized linear models (GLMs) were built for parameters which did not involve repeated measures: GLMs were built to understand the effect of treatment on (i) foraging behaviour (number of bites at worms) and (ii) the probability of not switching states (remaining solitary or remaining in a group). We checked the distribution of our data using the ‘fitdistr’ function [63] and as our data were closest to the normal (Gaussian) distribution we ran models assuming the Gaussian distribution of the data. Model comparisons were performed using ANOVA in ‘car’ package [61] and *post hoc* paired tests (Tukey’s *post hoc* HSD test using the ‘multcomp’ package [64]) were performed for comparing the effects of factors that were significant. Spearman’s correlation tests were then performed to analyse the relationship between their median position index and standard deviation from the median position index. Mean ± s.e. values have been reported throughout the paper. Two-tailed *p* ≤ 0.05 were considered significantly different.

## 3. Results

### 3.1. Shoaling and foraging behaviour

**Figure 1.**
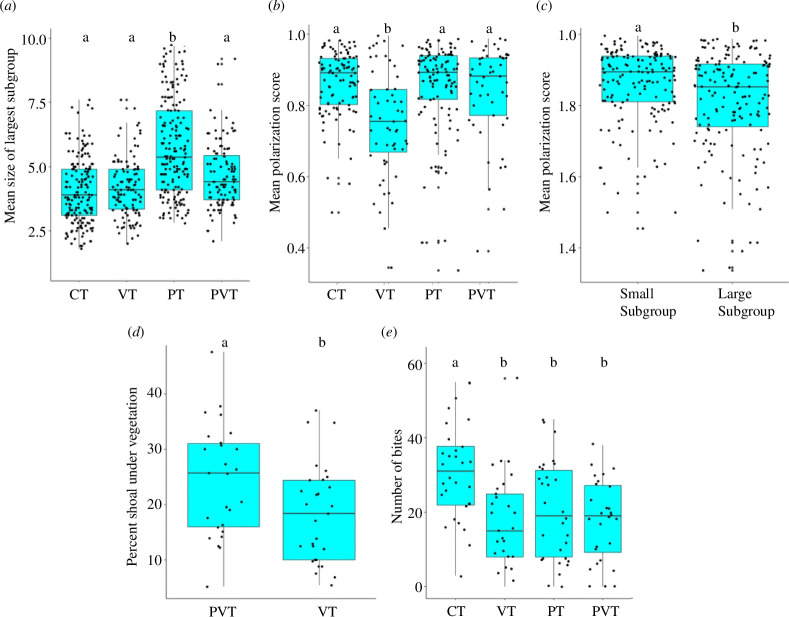
Box-and-whisker plots across treatments representing: (*a*) the mean size of largest subgroup, (*b*) the mean polarization score across treatments, (*c*) the mean polarization score across small and large subgroups, (*d*) the percent shoal under vegetation in the presence and absence of predator cues and (*e*) number of bites at worms. Each dot represents the average over one session of four sessions per group (*a–c*) or the average over all sessions (*d*) or the frequency of a unique group (*e*). The different letters (‘a’ and ‘b’) placed above the boxes represent significant differences between the treatments. The letters ‘ab’ indicate that the treatment is comparable to treatments ‘a’ and ‘b’. CT, control treatment; PT, predator treatment; PVT, predator vegetation treatment; VT, vegetation treatment. Comparisons were performed using Tukey’s HSD test (sample size: mean size of largest subgroup, mean polarization score: *N*_CT_ = *N*_PT_ = 45 shoals; *N*_VT_ = *N*_PVT_ = 30 shoals; *p* < 0.05).

The GLMM revealed that the mean largest subgroup size was significantly impacted by treatment (Wald type IIχ^2^ = 163.65, d.f. = 3, *p *< 0.001) and that the mean largest subgroup size was comparable across sessions (Wald type IIχ^2^ = 1.79, d.f. = 4, *p* = 0.77; table 1*a*). The mean largest subgroup size of PT shoals (mean = 5.72 ± 0.13) was significantly greater than the mean largest subgroup size of CT (mean = 4.06 ± 0.09), VT (mean = 4.18 ± 0.10) and PVT (mean = 4.64 ± 0.12) shoals (Tukey’s test results: CT versus PT: *Z* value = 11.24, *p *< 0.001; VT versus PT: *Z* value = −10.09, *p *< 0.001; PT versus PVT: *Z* value = −7.76, *p *< 0.001; figure 1*a*).

**Table 1a.** Results of the GLMM for predicting the effect of treatment and session on mean largest subgroup size. Model: mean size of largest subgroup ~ session + treatment + (1|shoalid).

coefficients				
	estimate	s.e.	*t* value	Pr (>|*t*|)
(intercept)	<0.001	<0.001	13.36	<0.0001
session 1	<0.001	<0.001	−0.01	0.99
session 2	<0.001	<0.001	0.64	0.52
session 3	<0.001	<0.001	0.3	0.76
session 4	<0.001	<0.001	0.19	0.85
PT	<0.001	<0.001	11.23	<0.001
PVT	<0.001	<0.001	3.2	<0.01
VT	<0.001	<0.001	−0.01	0.99

The GLMM revealed that the mean polarization score was significantly impacted by treatment (Wald type IIχ^2^ = 57.77, d.f. = 3, *p *< 0.001), size of subgroup (Wald type IIχ^2^ = 15.38, d.f. = 1, *p *< 0.001) and session (Wald type IIχ^2^ = 25.46, d.f. = 4, *p *< 0.001; table 1*b*). The mean polarization score of VT shoals (0.75 ± 0.02) was significantly lower than the mean polarization score of CT (mean = 0.86 ± 0.01), PT (mean = 0.85 ± 0.01) and PVT (mean = 0.83 ± 0.01) shoals (Tukey’s test results: CT versus VT: *Z* value = −6.79, *p *< 0.001; VT versus PT: *Z* value = −7.19, *p *< 0.001; VT versus PVT: *Z* value = −5.14, *p *< 0.001; figure 1*b*). Small subgroups were more polarized (0.86 ± 0.01) than large subgroups (mean = 0.80 ± 0.01) (Tukey’s test results: small subgroups versus large subgroups: *Z* value = 3.92, *p* < 0.0001; figure 1*c*) and the mean polarization score in session 4 (mean = 0.72 ± 0.02) was significantly lower than the mean polarization score in sessions 2 (mean = 0.82 ± 0.01) and 3 (mean = 0.86 ± 0.01) (Tukey’s test results: session 4 versus session 2: *Z* value = −4.47, *p *< 0.001; session 4 versus session 3: *Z* value = −4.10, *p *< 0.001).

**Table 1b.** Results of the GLMM for predicting the effect of treatment, session and subgroup size on mean polarization score. Model: mean polarization score ~ session + treatment + size+ (1|shoalid).

coefficients					
	estimate	s.e.	d.f.	*t* value	Pr (>|*t*|)
(intercept)	0.89	0.03	303.4	22.72	<0.0001
session 1	−0.07	0.03	273.49	−1.99	0.04
session 2	−0.04	0.03	271.47	−1.26	0.2
session 3	−0.05	0.03	273.25	−1.46	0.14
session 4	−0.13	0.03	272.85	−3.43	<0.001
PT	0.009	0.01	309.99	0.54	0.58
PVT	−0.02	0.02	298.32	−1.03	0.30
VT	−0.14	0.02	301.24	−6.79	<0.001
small	0.05	0.01	259.59	3.92	<0.001

The GLMM revealed a significant effect of treatment on percentage shoal under vegetation (Wald type IIχ^2^ = 3.96, d.f. = 1, *p* = 0.04; table 1*c*)—a significantly greater percentage of individuals were under vegetation in PVT (mean = 18.23 ± 1.58%) as compared with VT (mean = 24.31 ± 1.89%) (Tukey’s test results: VT versus PVT: *Z* value = −1.99; *p* = 0.04; figure 1*d*). Although statistically comparable, the time taken to emerge out of vegetation in the laboratory (in absence of cues) (mean = 181.56 ± 37.38 s) was less than that in the field in the presence of a variety of cues (mean = 301.43 ± 50.98 s) (Wilcox unpaired test results: *W* = 54, *p* = 0.07; electronic supplementary material, figure S2).

**Table 1c.** Results of the GLMM for predicting the effect of treatment on percent shoal under vegetation. Model: percent shoal under vegetation ~ treatment + (1|shoalid).

	estimate	s.e.	d.f.	*t* value	Pr (>|*t*|
(intercept)	23.69	1.92	52.87	12.34	<0.0001
VT	−4.94	2.48	29.64	−1.99	0.05

The GLM revealed a significant effect of treatment (Wald type IIχ^2^ = 22.78, d.f. = 3, *p* < 0.001) on number of bites at worms (table 1*d*): CT shoals bit significantly more worms (mean = 30.93 ± 2.31 bites) than shoals in VT (mean = 17.41 ± 2.32 bites), PT (mean = 20.33 ± 2.40 bites) or PVT (mean = 17.75 ± 2.01 bites) (Tukey’s test results: CT versus PT : *Z* value = −3.27, *p *< 0.01; CT versus PVT : *Z* value = −4.00, *p *< 0.001; CT versus VT: −4.14, *p *< 0.001; figure 1*e*).

**Table 1d.** Results of the GLM for predicting the effect of treatment on number of bites at prey (blood worms). Model: number of bites ~ treatment.

coefficients				
	estimate	s.e.	*t* value	Pr (>|*t*|)
(intercept)				
PT	−10.6	3.23	−3.27	0.001
PVT	−13.18	3.29	−4	<0.001
VT	−13.52	3.26	−4.14	<0.0001

### 3.2. Shoal dynamics within shoals receiving cues from a predator

**Figure 2.**
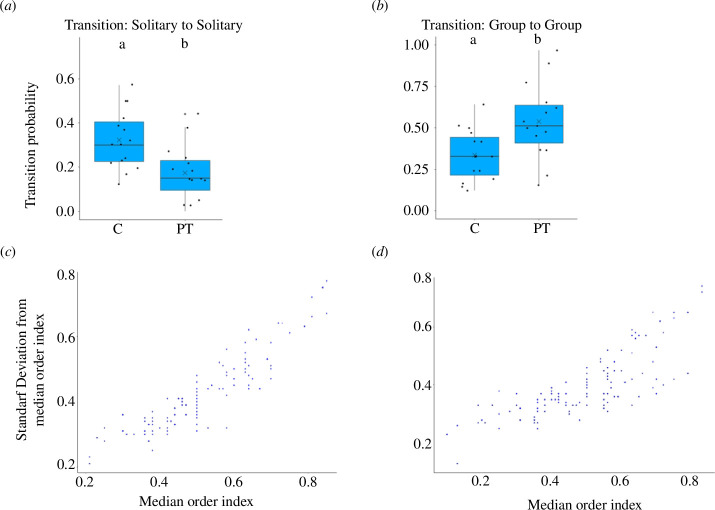
Shoal dynamics and deviation in individuals’ shoal position in control treatments and predator treatments. Box-and-whisker plots representing the probability of continuing to swim in: (*a*) solitary and (*b*) in group state for CT and PT. Each dot represents the mean probability of changing states by a unique group. The different letters (letters ‘a’ and ‘b’) placed above the boxes represent significant differences between the treatments. Comparisons were performed using Tukey’s HSD test (sample size: *N*_C_ = *N*_PT_ = 15 shoals; *p *< 0.05). Scatter plots representing the correlation between standard deviation from the median order index and the median order index in control treatments (*c*) and predator treatments (*d*). Each dot represents the median order index and the corresponding standard deviation from a median order index by an individual. The correlation was tested using Spearman’s correlation test (sample size: *N*_C_, *N*_PT_ = 150 individuals; *p *< 0.05).

The probability of continuing to swim solitary or continuing to swim in a group was dependent on the treatment (GLM results for (i) continuing to swim solitary: Wald type IIχ^2^ = 10.03, d.f. = 1, *p *> 0.01, table 2*a*; (ii) continuing to swim in group: Wald type IIχ^2^ = 8.13, d.f. = 1, *p *> 0.01; table 2*b*). The probability of continuing to be in a solitary state was significantly smaller among individuals in PT shoals (mean = 0.17 ± 0.03) as compared with individuals in CT shoals (mean = 0.32 ± 0.01); figure 2*a*. Correspondingly, the probability of continuing to be in a group was significantly higher among individuals in PT shoals (mean = 0.53 ± 0.05) as compared with individuals in CT shoals (mean = 0.33 ± 0.03) (figure 2*b*; Tukey’s test results in table 2*c*).

**Table 2a.** Results of the GLM for predicting the effect of treatment on mean transition probability for continuing to be in solitary state. Model: mean transition probability ~ treatment.

	estimate	s.e.	*t* value	Pr (>|*t*|)
(intercept)	0.32	0.03	9.7	<0.0001
PT	−0.14	0.04	−3.16	0.01

**Table 2b.** Results of the GLM for predicting the effect of treatment on mean transition probability for continuing to be in group state. Model: mean transition probability ~ treatment.

	estimate	s.e.	*t* value	Pr (>|*t*|)
(intercept)	0.33	0.04	6.72	<0.0001
PT	0.2	0.07	2.85	0.008

**Table 2c.** Tukey’s test results for continuing to be in a given state.

	estimate	s.e.	*Z* value	Pr (>|*Z*|)
continuing to be in solitary state				
PT - C	0.14	0.04	−3.17	<0.01
continuing to be in group state				
PT - C	0.2	0.07	2.85	<0.01

There was a strong correlation between the median order index and the standard deviation from the median order index for both treatments (CT: *R* ^2^ = 0.88; *p *< 0.0001; PT: *R* ^2^ = 0.83; *p *< 0.0001). Individuals in PT shoals and individuals in CT shoals showed a similar pattern with regards to their shoal position: individuals towards the front deviated less from their median position in the shoal as compared with other individuals (figure 2*c,d*).

## 4. Discussion

Our study reveals immediate changes in group-level characteristics among wild zebrafish (*Danio rerio*) shoals in response to two ecological factors. Test shoals exhibited considerable behavioural plasticity in the form of changes in shoal size, polarization and foraging behaviour in the presence of predation and/or vegetation. Furthermore, temporal analysis revealed that the tendency of individuals to remain in a group or to remain solitary is also strongly dependent on the presence of predator cues. Our study thus demonstrates that fish perceive various ecological factors and adjust their shoaling characteristics accordingly. It is likely that such immediate behavioural responses may be necessary for their survival in freshwater habitats.

### 4.1. Shoaling and foraging behaviour

Wild zebrafish shoals respond to predation and vegetation with considerable plasticity in shoaling and foraging. Increased group size and/or group cohesion to escape predators has been shown to occur in several species, including fish. Our findings on increased shoal size among zebrafish in the presence of predator cues are in consensus with previous studies on three-spined stickleback (*Gasterosteus aculeatus*), bluntnose minnows (*Pimephales notatus*), Pacific salmon (*Oncorhynchus* spp.), mosquitofish (*Gambusia affinis*), guppies (*Poecilia reticulata*), wild piranha (*Pygocentrus nattereri*) and fathead minnows (*Pimephales promelas*) [29,40,65–69]. Wild zebrafish shoals were highly polarized in open tanks (CT) (similar to sticklebacks (*Gasterosteus aculeatus*) [70]) and in predator cue treatments (PT and PVT), suggesting that predation odour and absence of vegetation are considered risky. Similar to barred flagtails (*Kuhlia mugil*) [71], smaller subgroups were more polarized as compared with larger subgroups.

A previous study conducted by us revealed that zebrafish shoals modulate anti-predator strategies based on the magnitude and kind of predator cues present: while shoals adhered to safety in numbers in the presence of visual or olfactory cues of a predator, individuals within shoals underwent increased freezing in the simultaneous presence of both cues from a predator [72]. The percent individuals under vegetation was significantly more in the presence of predator cues indicating that in the presence of vegetation, zebrafish shoals elicit a different kind of anti-predator strategy, wherein individuals take refuge or hide under vegetation when detecting the presence of a predator. The fact that shoals take longer to emerge from underneath natural vegetation in their natural habitats (as compared with laboratory studies lacking predator cues) could be due to the presence of additional predator/alarm cues in their natural habitat. Differences between natural vegetation and plastic aquarium plants in the experimental tank could be another explanation for this difference. This follow-up study extends our laboratory-based findings to field conditions.

These findings highlight the adaptability exhibited by shoals, illustrating how they modify their behaviour in the presence of aquatic vegetation and cues from predators. The present study also reveals that school responses to a given ecological variable may also depend on other ecological factors present. We speculate that future studies, incorporating additional factors such as turbidity and water flow, may reveal further modifications in predator avoidance strategies.

While shoals forage most effectively in the absence of vegetation and predator odour, a reduction in foraging in the presence of a predator is likely to be an anti-predator response [67,73,74]. Test shoals might choose a refuge (in the form of vegetation) over foraging in the open arena. In the possibility of zebrafish being visual foragers [75,76], the presence of vegetation might also obstruct access to visual information about the presence/location of food, likely reducing foraging efficiency. In their natural habitats, zebrafish feed on algae and zooplankton in the water [77] and therefore in such habitats, vegetation and their food sources are often not spatially separated. While this study clearly shows that vegetation acts as a refuge and may be useful in the context of predator avoidance, the same factor may obstruct useful information such as the presence of food sources.

### 4.2. Shoal dynamics within shoals receiving cues from a predator

Ecological factors like habitat, prey availability and predation strongly control fission–fusion dynamics among schooling fishes [78,79]. As in guppies (*Poecilia reticulata*), the analyses of fission–fusion dynamics revealed that in the presence of predator cues, the tendency of individuals to leave the largest subgroup declined significantly [80]. Thus, shoals exposed to predator cues not only exhibit larger subgroup sizes but also exhibit minimal changes in membership within these subgroups. In fish species such as the Atlantic cod (*Gadus morhua*), golden shiner (*Notemigonus crysoleucas*), mosquitofish (*Gambusia affinis*) and guppies (*Poecilia reticulata*) specific individuals (termed as leaders) consistently occupy the front of a shoal [81–83]. Our results reveal that regardless of the presence of predator odour, individuals towards the front of a zebrafish shoal showed lesser deviation from their positions as compared with individuals who followed. Therefore, we establish that individual leadership within shoals remains intact even as they display anti-predator responses.

Anti-predator strategies (or responses to other kinds of environmental changes) in animals are tightly linked to other ecological variables of a given habitat. While anti-predator responses have been studied across species, these do not address the influence of other ecological variables (such as vegetation cover, presence of refuge, type and abundance of co-occurring species) on anti-predator strategies. Our experiments demonstrate that shoals not only modify behaviour in response to predators but their anti-predator strategies also depend on the presence of vegetation. Future studies aiming to achieve comprehensive understanding of anti-predator tactics in different fish species (or in other animals) should consider additional ecological variables encountered. As our results show that vegetation is likely to enable shoals to avoid predators these findings can also aid in the formulation of conservation strategies. Wild zebrafish habitats are shared by a variety of other small freshwater species and face additional threats from invasive fish species, that can further increase predation pressure on zebrafish/other similar sized freshwater fishes. Vegetation along the edges of their habitats may aid such small freshwater fishes to escape predator attacks.

## Data Availability

Data for this study are available online [84]. Supplementary material is available online [85].
